# Does cellular senescence play an important role in the prognosis of sarcomatoid carcinoma of the pancreas?

**DOI:** 10.1186/s12957-021-02177-7

**Published:** 2021-03-16

**Authors:** Toshihisa Kimura, Tamotsu Togawa, Atsushi Iida, Sakon Noriki, Yasunori Sato, Takanori Goi

**Affiliations:** 1 Department of Surgery, National Hospital Organization, Tsuruga Medical Center, 33-1, Sakuragaoka, Tsuruga, Fukui, 914-0195 Japan; 2 Faculty of Nursing and Social Welfare Sciences, Fukui Prefectural University, 4-1-1 Kenjojima, Matsuoka, Eiheiji-cho, Yoshida-gun, Fukui, 910-1193 Japan; 3 Department of Human Pathology, Kanazawa University Graduate School of Medicine, 13-1, Takaramachi, Kanazawa, Ishikawa 920-8640 Japan; 4 First Department of Surgery, Faculty of Medicine, University of Fukui, 23-3, Shimoaizuki, Matsuoka, Eiheiji-cho, Yoshida-gun, Fukui, 910-1193 Japan

**Keywords:** Sarcomatoid carcinoma of the pancreas, Long-term survival, Epithelial-mesenchymal transition, cellular senescence, TGF-*β*

## Abstract

**Aim:**

Sarcomatoid carcinoma of the pancreas (SCP) is an extremely rare and aggressive disease with poor prognosis. We have already reported a rare case of SCP with long-term survival. In the present article, we investigate the underlying mechanisms of patient’s long-term survival from the point of view of cellular senescence which was examined in three SCP cases, including our reported case, using immunohistochemical analysis.

**Findings:**

The expressions of cellular senescence marker were observed in the sarcomatous component of the patient with long-term survival but not observed in the other patients with short- term survival. Thus, we can speculate that cellular senescence might play an important role in the reduction of the cell proliferative and metastatic activities of sarcomatous cells, leading to long-term patient survival.

**To the editor:**

Sarcomatoid carcinoma of the pancreas (SCP) is an extremely rare and aggressive disease with poor prognosis [1, 2]. The survival benefit of surgery for SCP remains uncertain, and no therapeutic strategies for SCP have been established. We have already reported a rare case of SCP with long-term survival [3]. In that article, we concluded that the low proliferative activity of sarcomatous cells might have played a role in the prolonged survival of the patient. In the present article, we investigate the underlying mechanisms of the low proliferative activity of sarcomatous cells from the point of view of cellular senescence, using immunohistochemical analysis, and we provide additional information from our previously published article. Epithelial-mesenchymal transition (EMT) is a plausible mechanism of SCP tumorigenesis [4]. In addition, transforming growth factor-β (TGF-β) is known to induce EMT [5, 6]. On the other hand, TGF-β also induces cellular senescence, which means that it exerts tumor-suppressive effects [7–9]. In the present study, the occurrence of the two important processes involved in cancer progression, EMT and cellular senescence, was examined in three SCP cases, including our reported case. The patient profiles were as follows. Case 1 is a 68-year-old male. The tumor size was 5 cm in maximum diameter. He underwent distal pancreatectomy and received 6 months of chemotherapy with gemcitabine after surgery. Remarkably, the patient survived for 11 years with no recurrence. Case 2 is a 68-year-old male. The tumor size was 4 cm in maximum diameter. He underwent distal pancreatectomy. He received neoadjuvant chemotherapy with TS-1(100 mg/day) for 4 weeks and received adjuvant chemotherapy with hepatic arterial infusion (details unknown). He died from progression of liver metastases at 18 months after surgery. Case 3 is a 65-year-old female. The tumor was huge and occupied the upper left abdomen. She received an intraperitoneal injection of CDDP (cisplatin) (25 mg). She was non-operable and died 2 months after the consultation. Multiple liver metastases and peritoneal metastases were found at autopsy.

Pathological examination showed that the three cancers were sarcomatoid carcinomas mainly composed of spindle cells. Notably, the cellular density of sarcomatous spindle cells was lower in case 1 than in case 2 and case 3, and the tumor of case 1 was associated with abundant fibrous stroma (Fig. 1). An immunohistochemical analysis was performed to examine the expression of phosphorylated Smad2/3 (pSmad2/3), Snail, fibronectin, γ-H2AX, p53, and p21 using paraffin-embedded tissue sections from the tumor. The expression of pSmad2/3 is regarded as a marker of the occurrence of intracellular signal transduction via TGF-β, and Snail is one of the major transcription factors involved in the regulation of TGF-β-mediated EMT. Fibronectin is an extracellular matrix component produced by cells as a consequence of the occurrence of EMT. In addition, γ-H2AX, p53, and p21 are commonly used as a marker of cellular senescence. Within tissue sections, the expression of these molecules was evaluated in the sarcomatous component. In all three cases, the expression of pSmad2/3, Snail, and fibronectin was observed in the sarcomatous components (Fig. 1). These results indicated that the sarcomatous component might be derived from the adenocarcinoma component via TGF-β-mediated EMT. In contrast, γ-H2AX, p53, and p21 were observed in the sarcomatous component of case 1 but not in case 2 or case 3 (Fig. 1). Thus, a portion of the cells in the sarcomatous component might have also undergone cellular senescence in case 1. In accordance with these results, the MIB-1 index of case 1 was 11%, while it was 19% in case 2 and 32% in case 3.

**Fig. 1:**
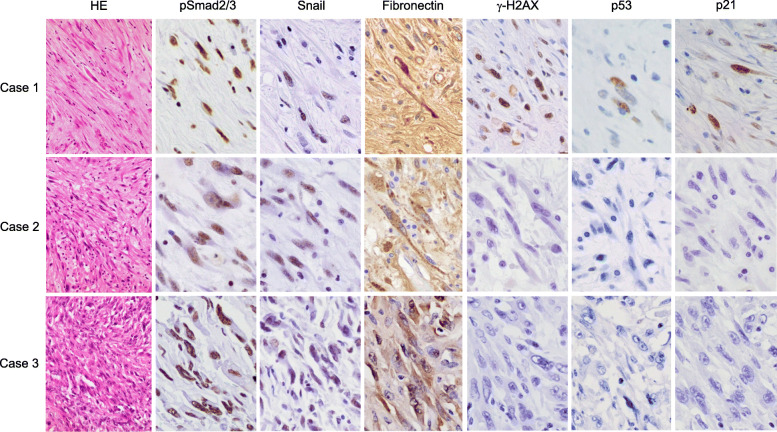
Immunohistochemical expression of EMT and cellular senescence-related molecules. The expression of pSmad2/3, Snail, and fibronectin was observed in the sarcomatous component of all cases. In contrast, γ-H2AX, p53, and p21 were observed in the sarcomatous component of case 1 but not in case 2 or case 3

It is generally known that SCP exhibits high rates of distant metastasis and local recurrence. In case 1, no distant metastasis or local recurrence was recorded during the 11 years after surgery. Although the exact mechanism explaining the patient’s long survival remains unclear, the low-grade malignant activity of the sarcomatous component is one possible explanation. The MIB-1 index of the sarcomatous cells in case 1 was 11%, which might be associated with the relatively low cellularity of sarcomatoid cells in this tumor compared with the other two cases (case 2 and case 3). EMT is critical for the invasion and metastasis of pancreatic ductal adenocarcinoma, and it may also contribute to the early-stage dissemination of cancer cells [5, 6]. TGF-β is a multifunctional cytokine that induces EMT through interactions with Smad2/3 and transcriptional factors such as Snail, and is reported to promote the formation of SCP [4]. In addition, TGF-β has another function, inducing the expression of p53 and p21 through the combined actions of Smad1/5/8 and Smad2, which subsequently leads to cellular senescence [**7**]. Cellular senescence is a state of irreversible cell cycle arrest provoked by persistent DNA damage, and it is a tumor-suppressive response that acts as a barrier to cancer development and progression [8]. Thus, TGF-β appears to have conflicting effects on cancer biology [9]. In case 1, the immunohistochemical expression of pSmad2/3 in carcinoma cells could be regarded as evidence that the intercellular TGF-β signaling pathway was activated within the tumor. The expression of Snail and fibronectin in sarcomatous cells further indicates the occurrence of EMT. Following EMT, the carcinoma cells might have invaded and progressed, and the resultant sarcomatous cells formed a large tumor with an abundant fibrous stroma via a mechanism mediated by the local production of extracellular matrix proteins. On the other hand, the sarcomatous cells in this case preferably expressed γ-H2AX, p53, and p21. The expression of p53 in sarcomatous cells might represent the induction of the active form of p53 rather than the abnormal accumulation of mutated p53 because the adenocarcinoma component did not express p53. These results suggest that TGF-β induced cellular senescence in sarcomatous cells through a pathway involving p53 and p21, reducing cell proliferative and metastatic activity. Interestingly, in case 2 and case 3, the patients received chemotherapy before the pathological specimens were obtained, and the markers indicative of cellular senescence were not detected in the specimens. This fact may indicate that cellular senescence is not induced by conventional chemotherapy. We can speculate that cellular senescence occurred more frequently than EMT in the sarcomatous component in case 1, leading to long-term survival of the patient. Our proposed molecular mechanism in case 1 is illustrated in Fig. 2.

**Fig. 2:**
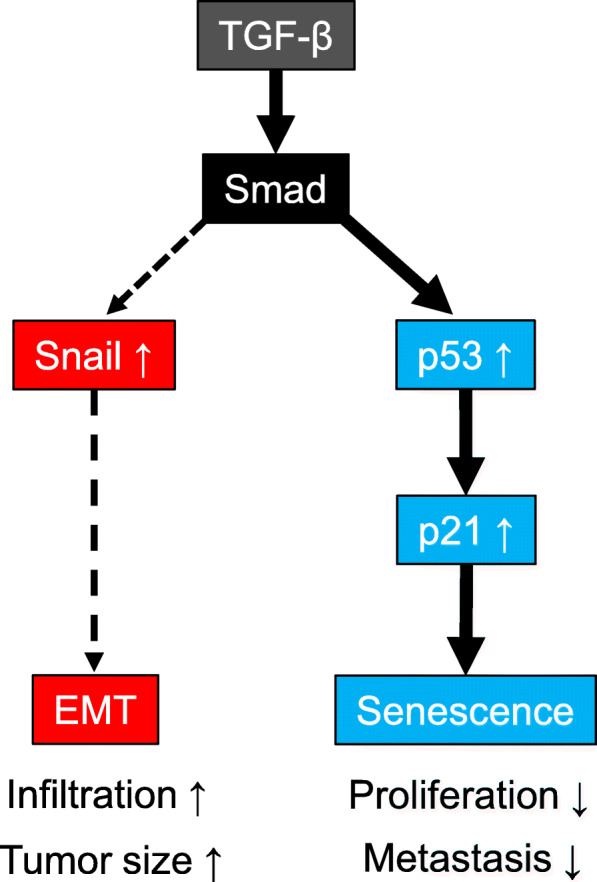
Proposed molecular mechanism accounting for the patient’s long-term survival. TGF-β has dual effects on the induction of both EMT and cellular senescence in pancreatic cancer cells, where cellular senescence might be predominant in case 1

Although the reasons for the prolonged survival in case 1 are not clear, we can speculate that cellular senescence induced by TGF-β might play an important role in the reduction of the cell proliferative and metastatic activities of sarcomatous cells. We are very aware of the limitation of our study: it includes an insufficient number of cases to determine the mechanisms explaining the long-term survival of the one patient with SCP. Therefore, the accumulation of additional data from more cases is necessary to further elucidate the mechanism underlying the development and progression of this type of carcinoma. On the other hand, our results may be of value with regard to strategies exploiting cellular senescence in SCP treatment because there is a possibility that TGF-βmay have beneficial effects on the inhibition of cancer progression by inducing cellular senescence through the pathway involving p53 and p21. Thus, it is mandatory to perform larger studies that investigate the relationship between the presence of cellular senescence and prognosis in SCP.

## Data Availability

All data generated or analyzed during this study are included in this published article.
